# Insecticidal Evaluation of Spinosad Against the Hide Beetle, *Dermestes maculatus* DeGeer (Coleoptera: Dermestidae)

**DOI:** 10.3390/insects17040375

**Published:** 2026-04-01

**Authors:** Shakila Khatun Bristy, Md. Nadim Aktar, Ataul Ahad, Md. Saiful Islam, Paraskevi Agrafioti, Christos G. Athanassiou, Md. Mahbub Hasan

**Affiliations:** 1 Department of Crop Science and Technology, Rajshahi University, Rajshahi 6205, Bangladesh; shakilabristy101@gmail.com (S.K.B.); nadim13579@gmail.com (M.N.A.); ataulahad5@gmail.com (A.A.); saifulcstru1002@yahoo.com (M.S.I.); 2 Laboratory of Entomology and Agricultural Zoology, Department of Agriculture, Crop Production and Rural Environment, University of Thessaly, Phytokou Str., 38446 Nea Ionia, Magnesia, Greece; athanassiou@uth.gr; 3 Department of Zoology, Rajshahi University, Rajshahi 6205, Bangladesh; mmhbgd@yahoo.com

**Keywords:** toxicity, spinosad, hide beetle, dried fish, bacterial pesticides

## Abstract

Spinosad, *Saccharopolyspora spinosa*, is widely used as a stored product protectant. However, its efficacy against the hide beetle *Dermestes maculatus* DeGeer (Coleoptera: Dermestidae), a major pest of dried animal-based materials, remains underexplored. In this study, we evaluated the contact and oral toxicities of spinosad across a range of concentrations applied directly to animal-derived substrates. Larval and adult mortality, along with probit parameters, were determined to assess susceptibility. Spinosad produced significant mortality in both life stages, with larvae exhibiting higher susceptibility under contact exposure, whereas adults showed increased susceptibility under feeding exposure. These patterns are consistent with prior reports of spinosad’s dual-action toxicity against stored-product pests. The larval contact and adult feeding exposures to spinosad were positively associated with higher doses, while adult contact and larval food exposures aligned with lower doses. The larval contact and adult ingestion routes drive the strongest toxic responses, whereas adult contact and larval feeding elicit weaker effects under the same doses. Overall, our results highlight spinosad as a promising tool for integrated pest management of hide beetles in animal-based storage systems.

## Simple Summary

Efficacy of Spinosad against the hide beetle *Dermestes maculatus* DeGeer (Coleoptera: Dermestidae), a key pest of animal-based commodities, remains insufficiently studied. In the present study, we evaluated the contact and oral toxicity of spinosad at different concentrations, applied directly to animal-derived substrates. The mortality of larvae and adults was assessed, and probit analysis was conducted to estimate susceptibility. Spinosad caused significant mortality in both life stages, with larvae being more susceptible under contact exposure and adults under feeding exposure. Toxicity increased with concentration, with larval contact and adult ingestion representing the most effective exposure routes. Overall, these findings indicate that spinosad has potential as an effective tool for the management of *D. maculatus* in stored animal products within integrated pest management (IPM) programs.

## 1. Introduction

Dried fish is a major source of protein in several developing countries [1]. Dried fish products frequently suffer from severe losses due to infestation by flesh flies (Sarcophagidae), beetles (*Dermestes*, *Cornestes*, and *Necrobia*), and mites (mostly *Lardoglyphus* and *Lyrophagus*) [2]. Moreover, dried fish are being contaminated by both insects and harmful insecticides, which is unacceptable for human consumption [3]. The losses are associated with a reduction in nutrient availability, leading to decreased consumer acceptability and market value, as well as to both quantitative and qualitative losses [4]. One of the most destructive insect pest species of dried fish is the hide beetle, *Dermestes maculatus* DeGeer (Coleoptera: Dermestidae). *Dermestes maculatus* is a species known for its role in the decomposition of animal remains and its presence as a pest in museums, stored products, and animal-based materials (such as skins, furs, and dried fish). Effective management of this beetle is important, especially in forensic entomology, museums, and food storage facilities [3,5,6,7].

In contrast with other stored product insect species, the data available for the methods that can be used with success for the control of this species are extremely limited, especially in bioassays where insecticides are applied directly on the commodity. Regarding fumigation, Athanassiou et al. [8] found that *D. maculatus* was susceptible to phosphine, with the egg stage exhibiting the highest level of tolerance. In the case of contact insecticides, it was found that this species was generally more tolerant than other stored product beetle species [9,10]. Nevertheless, most of these data are mostly focused on surface treatments, while there is still inadequate information on the direct application of contact insecticides on dried fish.

Spinosad, an insecticide that is based on metabolites of the bacterium *Saccharopolyspora spinosa* Mertz & Yao, has been proven effective against a wide range of stored product insects under both laboratory [11,12,13,14] and field conditions [15,16]. Currently, spinosad has been registered as a grain protectant in many parts of the world, at 1 mg (AI)/kg of grain. Its low mammalian toxicity makes Spinosad a viable solution over the use of traditional grain protectants, especially organophosphorous insecticides (OPs), which can be toxic to mammals. Subramanyam et al. [17] reported that spinosad was more effective than the OP chlorpyriphos-methyl against the lesser grain borer, *Rhyzopertha dominica* (F.) (Coleoptera: Bostrichidae), on wheat. On the other hand, Huang and Subramanyam [18] noted that spinosad was more effective than the OP pirimiphos-methyl against the rice moth, *Corcyra cephalonica* (Stainton) (Lepidoptera: Pyralidae). In addition, spinosad has been reported to control dermestid beetles and other pests associated with animal-derived materials, including dried meat, fish products, and museum specimens, suggesting that this insecticide may have potential for protecting protein-rich stored commodities [2,7]. These findings indicate that spinosad could be a promising option for the protection of stored non-grain products, particularly those of animal origin. Therefore, evaluating the efficacy of spinosad against the hide beetle on dried fish is important to determine its potential role in protecting animal-based stored commodities. Despite the fact that this insecticide has been extensively tested in different application scenarios, commodities, target and non-target species, and many other biotic or abiotic conditions [16], its efficacy against *D. maculatus* remains unexplored.

In this context, the present study aims to determine the contact and feeding effect of spinosad at different doses in larvae and adults of *D. maculatus* to illustrate the potential of using this insecticide in applications against this species. Moreover, our tests were carried out on treated shrimps, which are a commodity that has not been tested so far for the performance of spinosad.

## 2. Materials and Methods

### 2.1. Insect Rearing and Insecticide

The adults of *D. maculatus* were collected from the local dried fish market and subsequently cultured under controlled laboratory conditions. The colony was maintained on dried fish as the food medium at 27 ± 2 °C temperature and 65 ± 5% relative humidity in the Laboratory of Stored Product Pests, Department of Zoology, University of Rajshahi, Bangladesh, following the method described by Zakka et al. [19]. The insect colony was maintained under controlled conditions for ten successive generations prior to the commencement of the bioassays to ensure colony stabilization and adaptation. All experimental insects were obtained from this laboratory-reared population rather than from field-collected individuals. For standardization, only last instar larvae and 3–4-day-old adults were used in the experiments.

Laser^®^ was evaluated in this study, a commercial formulation containing 48% spinosad as a suspension concentrate (SC). This product was obtained from Dow AgroSciences, Athens, Greece. Spinosad is a naturally derived insecticide belonging to the spinosyn class, produced through the fermentation of *Saccharopolyspora spinosa*. It was prepared at different concentrations and applied in laboratory bioassays to evaluate its toxicity against *D. maculatus* adults under standardized conditions of 25 °C and 70% relative humidity. The rearing and bioassay procedures were conducted in an incubator chamber to ensure consistent environmental conditions and to minimize external influences on insect survival and development.

### 2.2. Bioassay I—Insecticidal Efficacy of Spinosad Applied on Adults and Larvae

Six concentrations of spinosad were evaluated, i.e., 0.25, 0.5, 0.75, 1, 2, and 4 ppm against larval and adult stages of *D. maculatus* [20]. The required concentrations were prepared by diluting appropriate amounts of spinosad in distilled water [21,22]. For the control, shrimp samples were treated with distilled water and handled identically to the treated groups.

Clean, empty plastic pots (190 mL) (RFL Company Limited, Dhaka, Bangladesh) were used for the bioassays. An aliquot of 0.5 mL of each spinosad concentration was applied to the pots using a micropipette, and the pots were left undisturbed for one hour to allow complete evaporation of the water. Then, the last ten instar larvae and adults (3–4 d old) of *D. maculatus* were placed in each pot separately (different series of pots for larvae and adults). For each combination (life stage and concentration), there were five replicates. The pots were then covered with gauze cloth and tied with rubber bands properly and were placed in an incubator (Wincom Company Limited, Shenzhen, China) set at 25 °C, 74% relative humidity, and a photoperiod of 16L:8D. Mortality was recorded after 2, 7, 15, and 30 days of exposure. After each exposure interval, a fresh shrimp was added to the pots to assess their survival. Fresh shrimp were provided after each observation interval to avoid microbial contamination and substrate degradation. This step was necessary to maintain consistent experimental conditions and to prevent potential confounding effects arising from changes in substrate quality that could influence insect response independently of the treatments. Dead larvae and adults were removed separately from the plastic pots after each exposure interval.

### 2.3. Bioassay II—Insecticidal Efficacy of Spinosad Applied on Diet

Clean and empty plastic pots (190 mL) were used in these bioassays, with 10 g of dried shrimps in each pot. The same concentrations and procedures as described in Bioassay I were followed for application to the diets. Then, 0.5 mL spinosad was applied using a micro pipette in the pot directly on the dried shrimp. After that, the last ten instar larvae and adults (3–4 d old) were placed separately in each pot, with a separate series of pots for each of the two life stages. The pots were covered with gauze cloth and tied with rubber bands properly to prevent insects from escaping. Five replicates were conducted for each combination. Then, they were placed in an incubator set at 25 °C, 74% relative humidity, and a 16L:8D photoperiod. The mortality of larvae and adults was counted after 2, 7, 15, and 30 days after the application of spinosad. Dead and alive individuals were removed after each exposure interval.

### 2.4. Statistical Analysis

Mortality was analyzed as cumulative mortality over the observation period. The observed mortality data were corrected using Abbott’s formula [23] to account for control mortality prior to analysis. The experiment followed a Completely Randomized Design (CRD). A two-way analysis of variance (ANOVA) using PROC GLM was conducted to evaluate the effects of spinosad concentration and exposure time (days), along with their interaction, on larval and adult mortality. Mean comparisons were performed using Tukey’s test at the 5% significance level. Furthermore, probit analysis (PROC PROBIT) was applied to the corrected cumulative mortality data to estimate lethal concentrations (LC_50_ and LC_99_) for each life stage [24].

## 3. Results

### Bioassay—Insecticidal Efficacy of Spinosad Applied on Larvae and Adults

**Figure 1:**
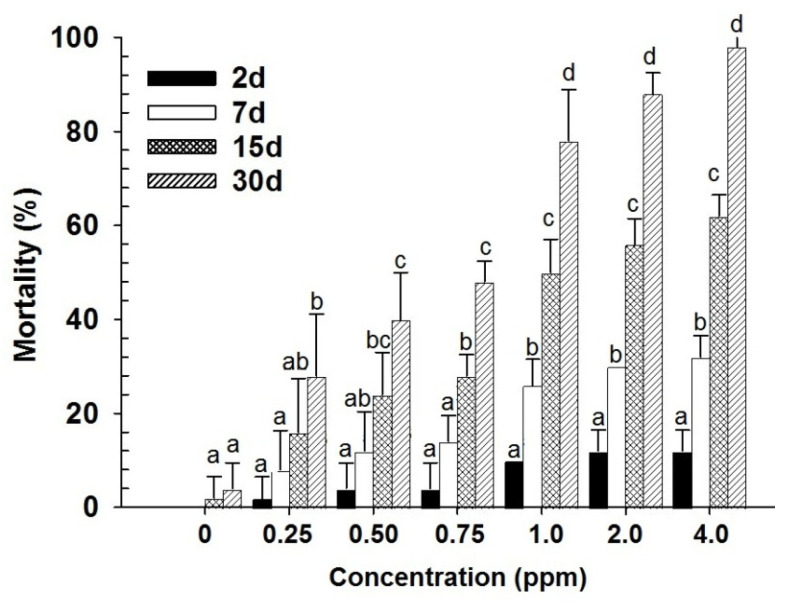
Mean mortality (% ±SE) of *D. maculatus* larvae exposed to spinosad by contact for different intervals (bars followed by the same letters within days are not significantly different; HSD test at 0.05).

**Figure 2:**
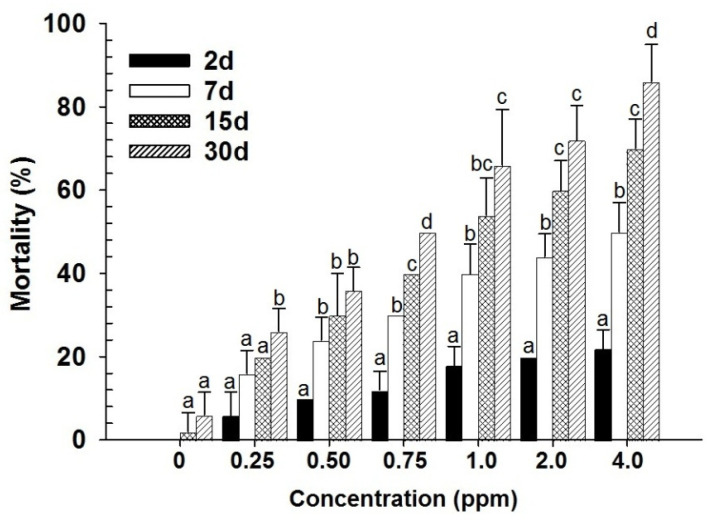
Mean mortality (% ±SE) of *D. maculatus* adults exposed to spinosad by contact for different intervals (bars followed by the same letters within days are not significantly different; HSD test at 0.05).

Contact toxicity of spinosad had a highly significant effect on mortality in last instar larvae of *D. maculatus* (dose: F_6,139_ = 245.06; days: F_3,139_ = 524.23; in both cases *p* < 0.001) (Figure 1). A significant interaction effect was also observed between dose and days (F_18,139_ = 28.71; *p* < 0.001), indicating that mortality patterns varied depending on both exposure duration and dose level. Probit analysis further revealed that larvae showed the lowest LC_50_ and LC_90_ values, confirming their higher susceptibility compared with adults (Table 1). Similar trends were evident in adult mortality (dose: F_6,139_ = 569.66; days: F_3,139_ = 958.15; dose × days: F_18,139_ = 50.60; in all cases *p* < 0.001) (Figure 2). However, adults consistently exhibited higher LC_50_ and LC_90_ values than larvae, indicating greater tolerance to the treatment (Table 1). Overall, mortality increased significantly with increasing concentrations and exposure time in both developmental stages. Larvae were more sensitive to the contact toxicity treatment, while adults required higher doses and longer exposure to achieve similar levels of mortality (Figure 1 and Figure 2).

**Figure 3:**
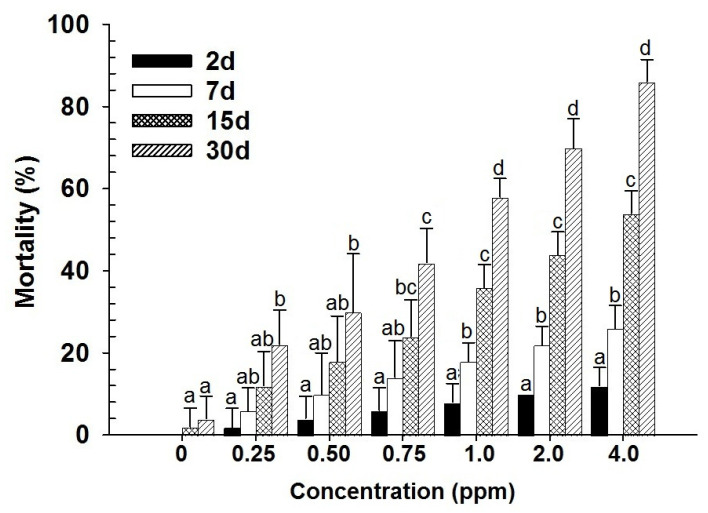
Mean mortality (% ±SE) of *D. maculatus* larvae exposed to spinosad through feeding for different intervals (bars followed by the same letters within days are not significantly different; HSD test at 0.05).

**Figure 4:**
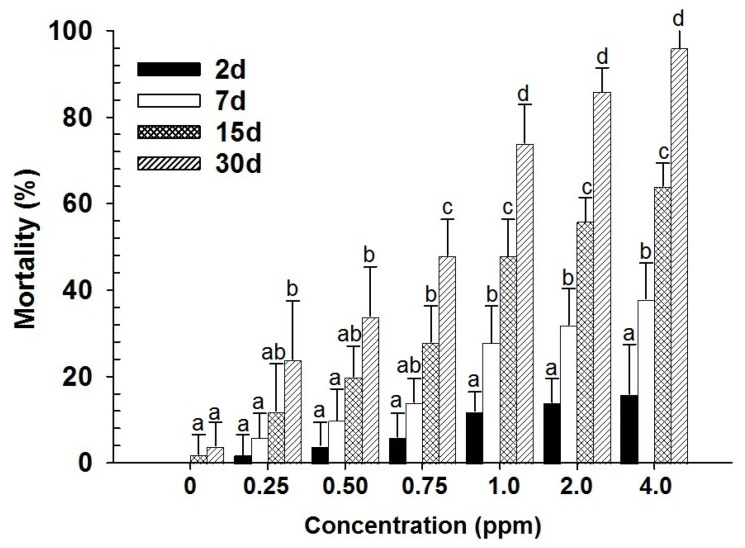
Mean mortality (% ±SE) of *D. maculatus* adults exposed to spinosad through feeding for different intervals (bars followed by the same letters within days are not significantly different; HSD test at 0.05).

With the contact toxicity, both main effects (dose and days) and their interaction (dose × days) were highly significant for both life stages (larvae–dose: F_6,139_ = 254.96; days: F_3,139_ = 553.20; dose × days: F_18,139_ = 30.96; adults–dose: F_6,139_ = 401.70; days: F_3,139_ = 685.44; dose × days: F_18,139_ = 38.83; in all cases *p* < 0.001) (Figure 3 and Figure 4). The significant interaction terms indicate that the rate of mortality depended not only on concentration but also on the duration of exposure, with mortality increasing progressively. Furthermore, probit analysis revealed that adults were more tolerant to spinosad than larvae in contact exposure, as shown by higher LC_50_ and LC_90_ values (Table 1). This suggests that larvae are more susceptible to spinosad in contact exposure, whereas adults require higher doses and longer exposure to achieve comparable levels of mortality. Overall, these findings confirm a stage-dependent difference in susceptibility.

The hierarchical cluster analysis (dendrogram) illustrates the similarity patterns of *D. maculatus* responses to spinosad exposure through different routes: contact and feeding for both larval and adult stages (supplementary file, Figure S1). Two distinct clusters emerged from the dendrogram. The first major cluster grouped larval contact and adult feeding, indicating a closer similarity in mortality or susceptibility under these two exposure routes. The second cluster comprised larval feeding and adult contact, suggesting that these treatments shared comparable toxicological impacts. The cluster heights reflect the relative dissimilarity, with larval contact and adult feeding showing the least divergence, while larval feeding and adult contact were relatively more distinct but still closely related.

Principal Component Analysis (PCA) showed that PC1 explained 99.5% of the total variation, indicating that the primary source of variability was the dose gradient across treatments. This confirms that dose is the dominant factor influencing insect responses in all exposure categories. Although the contribution of subsequent components was minimal, they were examined to assess any minor variation associated with life stage (larval vs. adult) and exposure route (contact vs. feeding). Thus, the PCA primarily serves to validate the overall response pattern and consistency across treatments rather than to reveal distinct multivariate structuring (supplementary file, Figure S2). In the biplot, the vectors corresponding to larval contact and adult feeding were projected toward the upper-right quadrant, closely aligning with the higher concentration points (1, 2, and 4 ppm). This pattern suggests that larval contact and adult feeding exposure are positively associated with the higher dose treatments and drive most of the observed variance along PC1. In contrast, adult contact and larval food were oriented toward the left and lower quadrants, nearer to the lower concentrations (0, 0.25, 0.5, and 0.75 ppm), indicating a negative or weaker association with increasing doses. Thus, PCA clearly separated treatments based on dose, with larval contact and adult feeding exposures clustering with higher concentrations, while adult contact and larval feeding exposures aligned with lower concentrations. This separation highlights the differential impact of exposure route (contact vs. feeding) and life stage (larva vs. adult) on response to the spinosad compound.

## 4. Discussion

Spinosad has been extensively evaluated for an extremely wide range of stored product beetles, but the present study is the first in which this insecticide was tested against *D. maculatus*. Based on our results, spinosad was very effective for both life stages tested here, at concentrations that are comparable to those that have been tested in earlier studies [16,21]. Previous studies have demonstrated that Spinosad is highly effective against several stored-product beetles, often achieving high mortality at relatively low doses and short exposure periods [13,15,16,22,25]. As expected, spinosad was more effective when applied to the diet of *D. maculatus* than when applied as a surface treatment.

We found that larvae were more susceptible than adults. This difference may be attributed to a combination of physiological and behavioral factors. Larvae exhibit continuous contact with treated surfaces due to their crawling behavior and limited mobility, which likely increases their exposure to contact insecticides. In the case of contact toxicity, we assume that larvae, when treated on the surface, were mostly exposed to the toxic effect of this insecticide, as compared with adults, who walk rather than crawl. Adults of this species have also been found to be very tolerant to other insecticides. For instance, Athanassiou et al. [9] found that this species was much more tolerant than other stored product beetle species to a combination of the pyrethroid beta-cyfluthrin and the neonicotinoid imidacloprid when applied on concrete. For instance, exposure of *D. maculatus* individuals in bags that were impregnated with the pyrethroid deltamethrin found that direct mortality was extremely low, as compared with other species, such as the warehouse beetle, *Trogoderma variabile* Ballion (Coleoptera: Dermestidae), the larger grain borer, *Prostephanus truncatus* (Horn) (Coleoptera: Bostrichidae) and *Rhyzopertha dominica* (F.) (Coleoptera: Bostrichidae). Our results are in accordance with these reports. Hence, it is likely that this tolerance of the adults of this species to various contact insecticides is a natural phenomenon, rather than an outcome of repetitive previous exposure, given that, in practice, spinosad has not been used extensively so far at the post-harvest stage of durable agricultural commodities.

Nevertheless, even in the case of larvae, mortality of *D. maculatus* was generally lower than that of what has been reported for other species at the same concentrations of spinosad [21]. In the present study, the estimated LC_50_ values for *D. maculatus* ranged from 0.27 ppm (larvae, contact) to 0.41 ppm (adults, contact), while LC_90_ values reached up to 0.81 ppm for adults. These values are generally higher than those reported for several common stored-product beetle species exposed to spinosad on grains, where effective control is often achieved at or below 1 ppm, frequently resulting in faster and higher mortality levels [15,21,22]. For example, larvae of another dermestid, khapra beetle, *Trogoderma granarium* Everts (Coleoptera: Dermestidae), were found to be tolerant to spinosad, as compared with other contact insecticides [26,27]. It is possible that dense setae covering the exoskeleton of dermestid larvae may provide a certain degree of protection, as these structures are known to function as a mechanical defense that can reduce external interactions [28,29]. As a result, increased larval survival or delayed larval mortality may increase the overall damage to the commodity. Similarly, delays in adult mortality may result in an increased progeny production before death and a rapid population rebound.

Although spinosad is currently registered as a grain protectant, we selected animal-based materials (shrimps) to be the treated substrate here, given that these are the usual commodities on which this species dominates. Moreover, our bioassays were based on a similar range of concentrations that have been tested in bioassays with other stored product beetle species as well as on grains [21]. We found that, despite some variations, mortality in these materials tested here was comparable to that on grains, suggesting that this insecticide may be a viable treatment for the protection of animal-based materials from stored product insects.

The clustering pattern suggests that spinosad exerts differential toxicological effects depending on both the developmental stage (larvae vs. adult) and the mode of exposure (contact vs. feeding). The similarity between larval contact and adult feeding implies that cuticular penetration in larvae and ingestion in adults may trigger comparable levels of mortality. Spinosad, a natural fermentation product of *Saccharopolyspora spinosa*, acts primarily through ingestion and secondarily by contact, leading to disruption of nicotinic acetylcholine receptors and GABA-gated chloride channels [28,29]. Thus, the ingestion pathway in adults and the contact pathway in larvae may overlap in terms of effective neurotoxic action.

Conversely, the grouping of larval feeding with adult contact reflects the potential differences in susceptibility due to physiological and behavioral traits. Hide beetle larvae are voracious feeders, and spinosad ingestion can result in rapid accumulation, leading to higher mortality rates [28]. In contrast, adult contact exposure is influenced by cuticular absorption efficiency and mobility, which may parallel the larval feeding route in terms of toxicological dynamics.

These findings are consistent with prior reports that spinosad is highly effective against stored-product pests through both ingestion and contact, though efficiency varies by insect species and life stage [21,29]. For *Dermestes* spp., the observed clustering highlights the importance of considering both developmental stage and exposure route when designing integrated pest management strategies. Specifically, targeting larvae through feeding substrates and adults via contact surfaces may provide synergistic control.

The PCA biplot revealed that nearly all the variation in our dataset was explained by the first principal component (Dim1 = 99.5%), with Dim2 contributing minimally (0.3%). The projection of vectors showed a clear separation by dose: larval contact and adult feeding exposures clustered in the upper-right quadrant, aligning with higher concentrations (1–4 ppm), whereas adult contact and larval feeding were oriented toward the left and lower quadrants near the lower concentrations (0–0.75 ppm).

This pattern agrees with previous studies demonstrating that life stage and exposure route strongly influence spinosad efficacy. Several authors have reported that larvae of stored-product pests are highly susceptible to spinosad through direct contact or ingestion, exhibiting pronounced lethal and sublethal effects such as delayed development, reduced pupation, and lower adult emergence [30,31]. Such responses would produce strong separation along a dose axis when larval-contact assays are included. Similar results have been observed in lepidopteran larvae [32,33] and mosquito larvae [34,35], who reported clear dose-dependent larvicidal activity of spinosad.

Our finding that adult (feeding) clustered with higher doses but adult (contact) did not reflect the results of Toews et al. [36], who showed ingestion exposure was generally more toxic than brief topical/contact exposure for several stored-product beetles. Differences in cuticle structure, behavioral avoidance, and mobility between larvae and adults may also explain the weaker association of adult contact with high doses [37]. In addition, studies by Elliott et al. [38] and Chayka and Boina [39] reported that ingestion or prolonged exposure to spinosad caused higher mortality than short-term contact in adult beetles, consistent with our PCA pattern.

Cross-stage effects of larval exposure are also well documented. For example, larval treatment with spinosad has been shown to reduce adult fecundity and longevity even when mortality at the larval stage was incomplete [40]. These carry-over effects could accentuate the association between larval contact and higher dose responses observed in our analysis.

Our results highlight two practical points. First, both life stage and exposure route must be considered when interpreting dose–response relationships. Second, application strategies should be designed to maximize exposure for the most vulnerable stage—here, larval contact and adult ingestion. However, because adult contact efficacy depends strongly on substrate, formulation, and exposure duration [34], control programs should be validated under the specific storage or field conditions of interest.

Finally, PCA is exploratory and does not establish causation. Future research should apply factorial designs to disentangle dose, life stage, and exposure route and measure both lethal and sublethal endpoints across stages [30,32]. Our PCA confirms that dose is the dominant source of variation in our data, while life stage and exposure route determine how that dose effect is expressed. These findings are consistent with published evidence showing strong larval susceptibility and the importance of ingestion exposure for adults in many species, and they also underscore the known context dependence of contact efficacy—highlighting the need for formulation- and context-specific evaluations before operational recommendations are made [34,36].

## 5. Conclusions

In conclusion, our results clearly indicate that spinosad can be effective for the control of the mobile life stages of *D. maculatus*, especially against the larval stages. Both application scenarios tested here proved the potential of this insecticide against this species. Nevertheless, extensive experimentation on a wider range of animal-based substrates will strengthen the knowledge for its use against *D. maculatus* and encourage its registration and use for this purpose.

## Figures and Tables

**Table 1:** Probit analysis for LC_50_, LC_90_, and LC_95_ (confidence intervals) of larvae and adults of *D. maculatus* treated with spinosad as a contact and feeding method.

Stages	Insect Used	LC_50_ (ppm)	LC_90_ (ppm)	LC_95_ (ppm)	Intercept ± SE	Slope ± SE	Chi (df)
Larvae (contact)	350	0.27 (0.25–0.28)	0.37 (0.33–0.47)	0.67 (0.46–3.77)	1.90 ± 0.29	2.98 ± 0.64	1.18
Larvae (feeding)	350	0.43 (0.38–0.47)	1.09 (0.73–10.37)	12.93 (1.92–14.09)	1.10 ± 0.22	2.20 ± 0.55	1.87
Adults (contact)	350	0.41 (0.38–0.44)	0.81 (0.62–2.45)	3.99 (1.27–5.03)	1.24 ± 0.23	2.47 ± 0.56	3.28
Adults (feeding)	350	0.34 (0.32–0.36)	0.48 (0.43–0.66)	0.94 (0.62–5.72)	1.69 ± 0.26	3.05 ± 0.60	4.97

## Data Availability

The original contributions presented in this study are included in the article/Supplementary Materials. Further inquiries can be directed to the corresponding author.

## Supplementary Materials

The following supporting information can be downloaded at: https://www.mdpi.com/article/10.3390/insects17040375/s1.
