# Supplementary material for: Insecticidal Evaluation of Spinosad Against the Hide Beetle, Dermestes maculatus DeGeer (Coleoptera: Dermestidae)
Source: Insects. 2026 Apr 1;17(4):375. doi: 10.3390/insects17040375 (PMC13115977; doi:10.3390/insects17040375)
Supplement: Supplementary file 1 [file insects-17-00375-s001.zip › insects-4208537-supplementary.pdf]

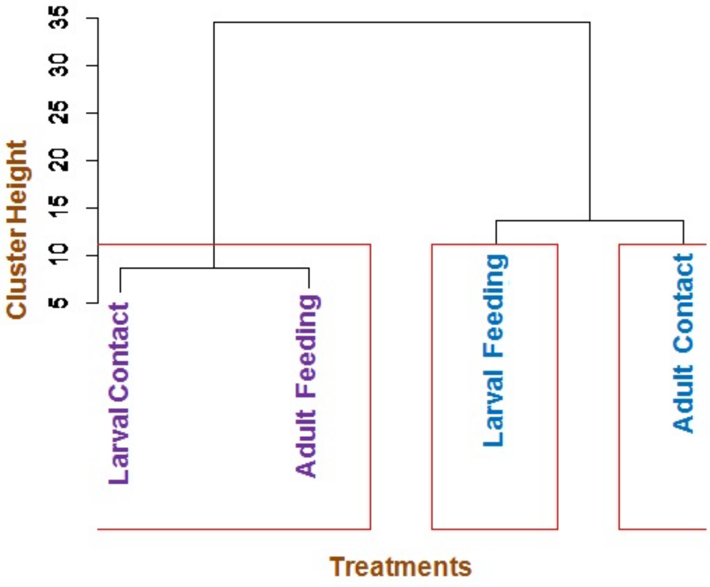

Figure S1. Dendrogram illustrating the clustering of larval and adult mortality of *D. maculatus* following contact and feeding exposure to spinosad.

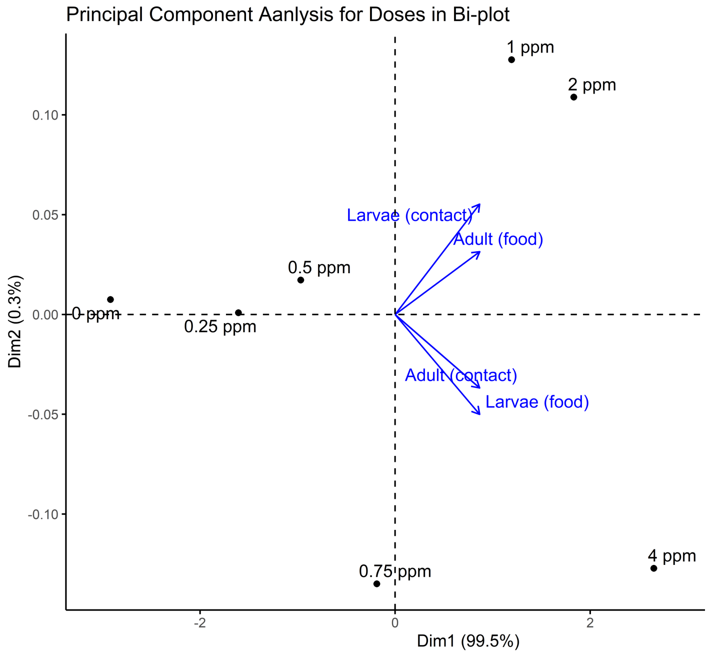

Figure S2. PCA biplot showing the mortality of larvae and adults of *D. maculatus* exposed to spinosad as contact and feeding methods. Arrows indicate the direction and strength of variables contributing to variance. Treatments closer together exhibit similar effects on mortality.
